# Shared spatiotemporal category representations in biological and artificial deep neural networks

**DOI:** 10.1371/journal.pcbi.1006327

**Published:** 2018-07-24

**Authors:** Michelle R. Greene, Bruce C. Hansen

**Affiliations:** 1 Neuroscience Program, Bates College, Lewiston, ME, United States of America; 2 Department of Psychological and Brain Sciences, Neuroscience Program, Colgate University, Hamilton, NY, United States of America; Harvard University, UNITED STATES

## Abstract

Visual scene category representations emerge very rapidly, yet the computational transformations that enable such invariant categorizations remain elusive. Deep convolutional neural networks (CNNs) perform visual categorization at near human-level accuracy using a feedforward architecture, providing neuroscientists with the opportunity to assess one successful series of representational transformations that enable categorization *in silico*. The goal of the current study is to assess the extent to which sequential scene category representations built by a CNN map onto those built in the human brain as assessed by high-density, time-resolved event-related potentials (ERPs). We found correspondence both over time and across the scalp: earlier (0–200 ms) ERP activity was best explained by early CNN layers at all electrodes. Although later activity at most electrode sites corresponded to earlier CNN layers, activity in right occipito-temporal electrodes was best explained by the later, fully-connected layers of the CNN around 225 ms post-stimulus, along with similar patterns in frontal electrodes. Taken together, these results suggest that the emergence of scene category representations develop through a dynamic interplay between early activity over occipital electrodes as well as later activity over temporal and frontal electrodes.

## Author summary

We categorize visual scenes rapidly and effortlessly, but still have little insight into the neural processing stages that enable this feat. In a parallel development, deep convolutional neural networks (CNNs) have been developed that perform visual categorization with human-like accuracy. We hypothesized that the stages of processing in a CNN may parallel the stages of processing in the human brain. We found that this is indeed the case, with early brain signals best explained by early stages of the CNN and later brain signals explained by later CNN layers. We also found that category-specific information seems to first emerge in sensory cortex and is then rapidly fed up to frontal areas. The similarities between biological brains and artificial neural networks provide neuroscientists with the opportunity to better understand the process of categorization by studying the artificial systems.

## Introduction

Categorization, the act of grouping like with like, is a hallmark of human intelligence. Scene categorization in particular is incredibly rapid [1,2] and may even be automatic [3]. Despite the importance of this problem, little is known about the temporal dynamics of neural activity that give rise to categorization. A common conceptual framework in the visual neuroscience community considers representations in each visual area as a geometric space, with individual images represented as points within this space [4,5]. According to this account, early visual processing can be described as a tangled geometric surface in which individual categories cannot be easily separated, and that over the course of processing, the ventral visual stream disentangles these manifolds, allowing for categories to be distinguished [6,7]. Although this view has provided a useful descriptive framework, we still know little about how this disentangling occurs because it has been difficult to examine the representations at each stage of processing.

In a parallel development, work in computer vision has resulted in the creation of deep convolutional neural networks (CNNs) whose categorization abilities rival those of human observers [8]. Although CNNs do not explicitly seek to model the human visual system, their architectures are inspired by the structure of the human visual system [9]: like the human visual system, they are arranged hierarchically in discrete representational layers, and they apply both linear and nonlinear operations across the whole of visual space. As CNNs are explicitly trained for the purpose of visual categorization, and as they achieve near human-level performance at this task, they provide neuroscientists with an unprecedented opportunity to interrogate the types of intermediate level representations that are built en route to categorization. Indeed, there is a growing literature detailing the similarities between aspects of CNNs and activity in biological brains [10–17].

Of particular interest to the current study is the correspondence between the stages of processing within a CNN and the temporal order of processing in the human brain, as assessed with M/EEG. Studies have demonstrated that the brain activity elicited by individual images can be predicted by the CNN [12,18] and that upper layers of the CNN can predict semantically-relevant spatial properties such as overall scene volume [19]. However, these studies pool across all sensors at a given time point, discarding potentially informative scalp patterns. While this capitalizes on the fine temporal scale of M/EEG, a complete understanding of the neural dynamics of scene understanding requires the characterization of information flow across the cortex. Additionally, key questions remain open, including understanding the development of scene category membership and how intermediate stages of visual representation allow for these complex abstractions to take place.

Therefore, the goal of the current study is to assess the extent to which sequential representations in each layer of a pre-trained deep convolutional neural network predict the sequential representations built by the human brain using high-density time-resolved event-related potentials (ERPs). Previewing our results, we show a spatiotemporal correspondence between sequential CNN layers and the order of processing in the human visual system. Specifically, earlier layers of the CNN correspond better to early time points in human visual processing and to electrodes over occipital and left temporal scalp regions. Later layers of the CNN correspond to later information and best match electrodes in the frontal half of the scalp, as well as over the right occipitotemporal cortex. The correspondence between computer models and human vision provides neuroscientists with the unique opportunity to probe intermediate-level representations *in silico*, allowing for a more complete understanding of the neural computations generating visual categorization.

## Results

**Fig 1:**
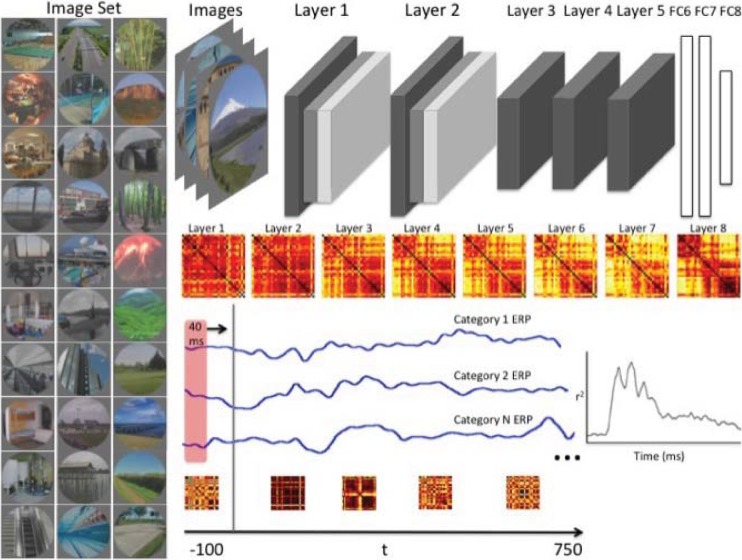
The image set consisted of 75 images from each of 30 scene categories. Example images are shown on the left. Activations for each of the eight layers of a pre-trained deep convolutional neural network (CNN) were extracted for each of the 2250 images. We averaged across exemplars to create 30x30 representational dissimilarity matrices (RDMs) for each layer of the CNN. For each participant, and for each of the 256 electrodes, we ran a 40 ms sliding window from the 100 ms before stimulus presentation, and running through the 750 ms where image was on screen. At each time point, a 30x30 RDM was created from the average voltage values at the given electrode and compared to each layer of the CNN using correlation. Note: although full matrices are shown, only the lower triangle was used in analysis.

We recorded high-density 256-channel EEG while 13 human participants viewed 2,250 photographs from 30 scene categories for 750 ms each while engaged in a three-alternative forced choice (3AFC) task. An additional 15 participants viewed the same images with the same procedure for 500 ms to serve as a full internal replication. Our approach is graphically illustrated in Fig 1. To compare scene category representations in a pre-trained (Places-205 with 8-layer “AlexNet” architecture [20]) deep convolutional neural network (CNN) to those of human observers, we used the representational similarity analysis (RSA) framework [5]. This allowed us to directly compare models and neuroelectric signals by abstracting both into a similarity space.

### CNN decoding results

**Fig 2:**
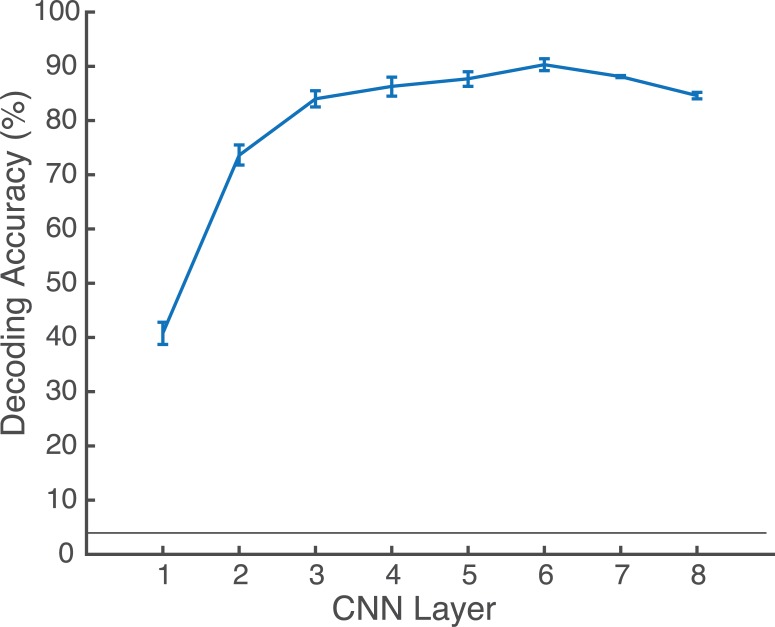
Category decoding accuracy for each layer of the CNN. Error bars reflect 95% confidence intervals. Horizontal line indicates chance level (1/30).

In order to understand the potential contributions of each CNN layer to category-specific neural responses, we assessed the extent to which each CNN layer contained decodable category information. All layers contained above-chance information for decoding the 30 scene categories (see Fig 2). The classification accuracy ranged from 40.8% in the first layer (Conv1) to 90.3% correct in the first fully-connected layer (FC6). Somewhat unexpectedly, this is higher accuracy than we observed in the top layer (FC8: 84.6%). It is also noteworthy that this level of classification is higher than the 69–70% top-1 classification accuracy reported in [20]. This is likely due to the fact that some of the images in our dataset were from the training set for this CNN. In sum, we can expect to see category-specific information in each of the eight layers of the CNN, with maximum category information coming from the fully connected layers, peaking in the sixth layer.

### Time-resolved encoding results

**Fig 3:**
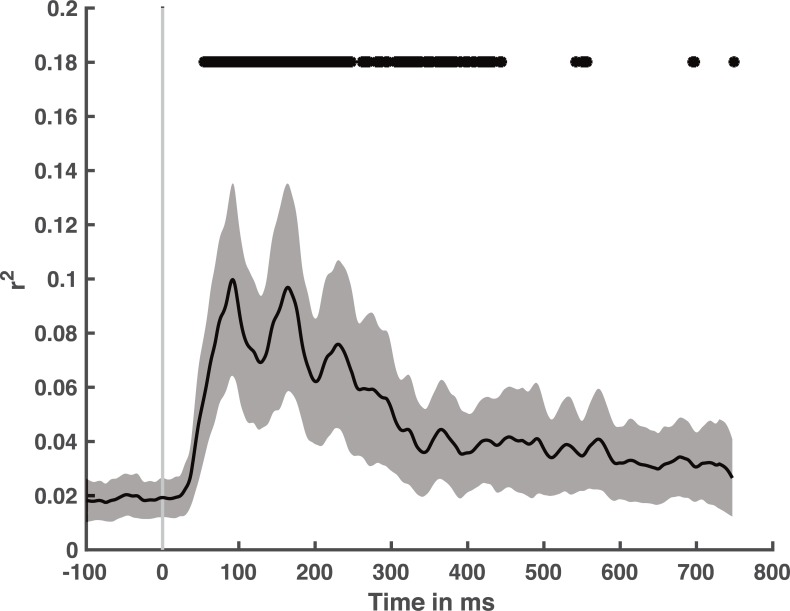
Variance explained over time for all eight CNN layers together for the average of all 256 electrodes. Black line indicates mean with shaded gray region reflecting 95% confidence interval. Top line indicates statistical significance.

To get an overall picture of human/CNN correspondence, we examined the extent to which the set of all CNN layers have explanatory power for visual ERPs. Our data-driven clustering method identified five groups of electrodes. We averaged ERPs within each cluster and then examined the variance explained by all layers of the CNN within that cluster’s representational dissimilarity matrices (RDMs) over time. For each cluster, we examined the onset of significant explained variance, the maximum explained variance, and the latency of maximum explained variance. We observed no statistically significant differences between the clusters for onset (F(4,60)<1), maximum explained variance (F(4,60) = 1.11, p = 0.36), nor latency at maximum (F(4,60)<1). Thus, we show the average of all 256 electrodes in Fig 3. We found that the CNN could predict neuroelectric activity starting at 54 ms after scene presentation (55 ms for internal replication set), and it achieved maximal predictive power 93 ms after stimulus onset (75 ms for internal replication set).

To assess how much of the explainable ERP variance was captured by the CNN, we estimated the noise ceiling using a method suggested by [21]. We found that the maximum explainable variance to be 48–54% (internal replication set: 45%-48%). Therefore, the average maximum *r*^2^ of 0.10 across all electrodes does not account for all variability in this dataset. As CNNs are exclusively feedforward models, the remaining variability to be explained may reflect the role of feedback in human scene categorization [22,23].

**Fig 4:**
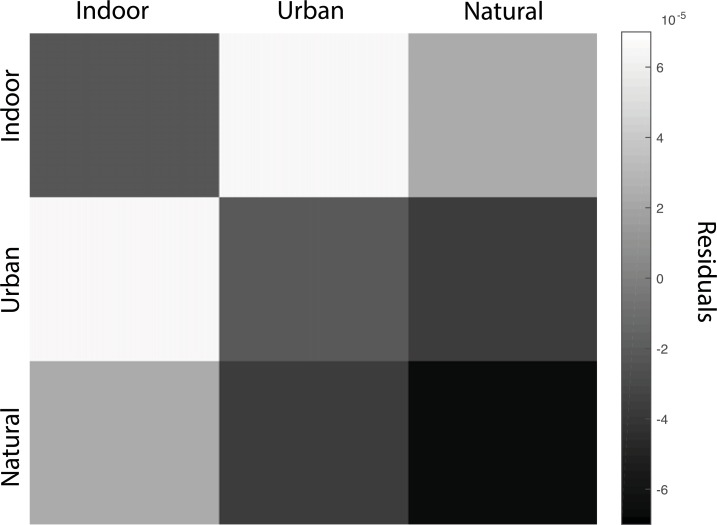
Residuals as a function of superordinate-level scene category for all eight layers of the CNN between 50 and 250 ms post-stimulus onset. Negative values indicate over-prediction of neural similarity, while positive values indicate under-prediction of neural similarity.

In order to gain additional insight into the relative successes and failures of the CNN as a model for visual scene categorization, we examined the regression residuals between 50 and 250 ms after stimulus onset. We averaged the residuals over participant, resulting in a 30x30 matrix. We then averaged over superordinate category (indoor, urban outdoor, and natural outdoor) in order to visualize broad patterns of CNN/neuroelectric difference. As seen in Fig 4, we observed negative residuals among and between the two outdoor superordinate categories. This pattern indicates that the CNN predicted *more* similarity between these categories than what was observed neurally. In other words, that the human category representations of outdoor categories are more distinct compared to the predictions made by the CNN. By contrast, the residuals between indoor and urban categories were positive, suggesting that the CNN had a finer-grained representation of these category differences compared to the human brain. The residuals for the replication set demonstrated the same broad pattern (see Supporting Material).

**Fig 5:**
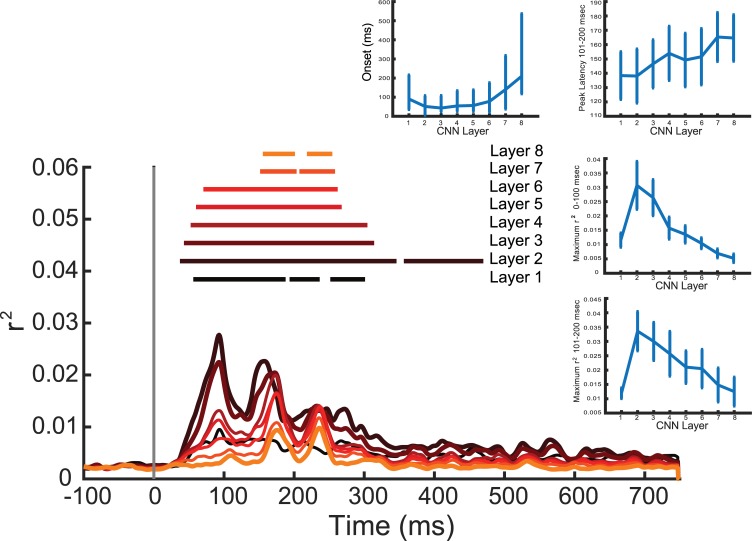
Main plot: Variance explained in each of the eight layers of the CNN taken alone. Each waveform represents the average of all 256 electrodes. Lowest layers are in darker colors, and horizontal lines represent statistical significance. Insets, clockwise from top: (1) Onset of statistically significant encoding as a function of CNN layer; (2) maximum variance explained between 101 and 200 ms as a function of CNN layer; (3) maximum variance explained between 0 and 100 ms as a function of CNN layer; (4) maximum variance explained between 101 and 200 ms as a function of CNN layer. Taken together, we can see that early CNN layers explain more variance in ERP patterns, and do so earlier than later CNN layers. All error bars reflect +/- 1 SEM.

In order to examine the correspondence between the processing stages of the CNN and the representational stages in human visual processing, we next examined the variance explained by each individual CNN layer. Averaged across all 256 electrodes, we observed a correspondence between the onset of explained variability and CNN layer, with earlier layers explaining ERP variability earlier than later layers (r = 0.38, p<0.001 for the main set and r = 0.40, p<0.001 for the replication set, see Fig 5). Additionally, we observed a negative relationship between CNN layer and explained variability, with earlier layers explaining more ERP variability than later layers. This effect was pronounced in the first 100 ms post-stimulus (r = -0.51, p<0.0001 for the main set and r = -0.49, p<0.0001 for the replication set), and was also observed between 101 and 200 ms (r = -0.26, p<0.005 for the main set and r = -0.23, p<0.05 for the replication set). A one-way ANOVA found a significant difference in the onset of significant explained variability as a function of CNN layer (F(7,96) = 5.46, p<0.0001) for the main dataset and F(7,112) = .4.81, p<0.001 for the replication set) Additionally, a one-way ANOVA revealed significant differences in the maximum explained variability across CNN layers: F(7,96) = 15.7, p<0.0001 for the main dataset and F(7,112) = 10.1, p<0.0001 for the replication set).

**Fig 6:**
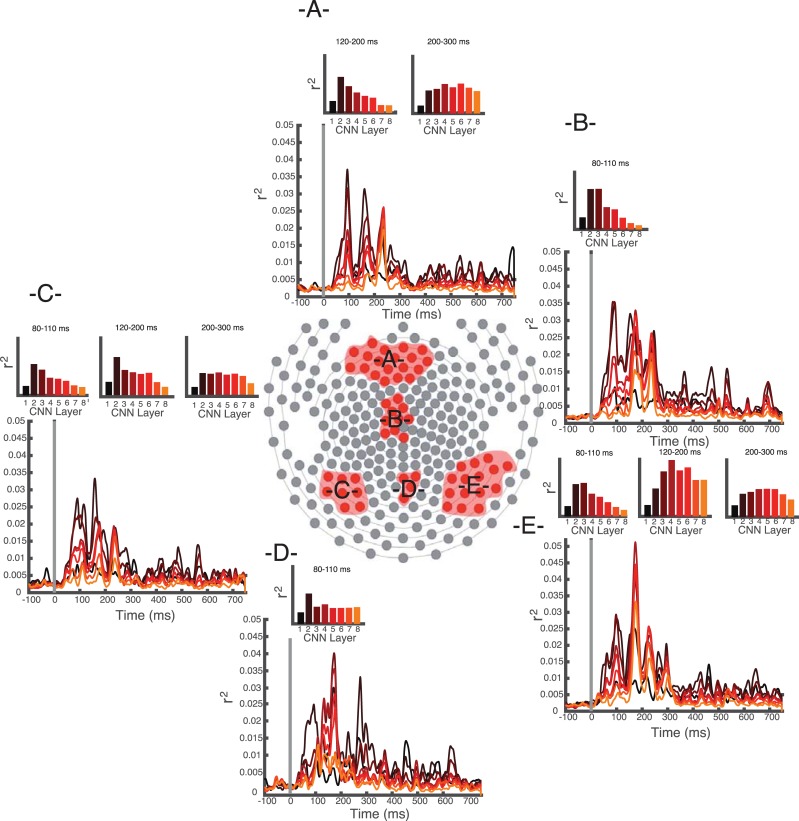
Encoding time course for each of the five identified clusters. For each plot, main graph shows variability explained over time. Bar charts show maximum explained variability for each of eight CNN layers in the post-stimulus time window indicated above.–A- Frontal cluster.–B- Central cluster.–C- Left occipitotemporal cluster.–D- Central occipital cluster.–E- Right occipitotemporal cluster.

A chief advantage of conducting the encoding analyses at each electrode, rather than collapsing across all sensors as has been done in previous work [12,18], is that we are able to examine the spatiotemporal patterns of explained variability rather than just temporal. Using data-driven electrode clustering (see Methods), we identified five groups of spatially contiguous electrodes with differing voltage patterns. The clustering took microvolt patterns in the topographic maps at each time point as input and tested for spatially continuous groups of electrodes that had similar microvolt patterns. We tested these against a chance level defined by a baseline topographic map. In each group, we examined the maximum explained variability for each layer of the CNN. As shown in Fig 6, we observed a striking dissociation. Central occipital electrodes were better explained by the earlier layers of the CNN at all time points (Spearman’s rank-order correlation between layer and maximum explained variance: (rho = -0.64, t(12) = -13.5, p<0.0001 for main set; rho = -0.68, t(14) = -13.3, p<0.0001 for replication set). A similar trend was seen in left occipitotemporal electrodes for time points before 200 ms in the main dataset, with a trend in the replication dataset (80–110 ms: rho = -0.53, t(12) = -6.31, p<0.0001 for main set; rho = -0.78, t(14) = -10.3, p<0.0001 for replication set; 120–200 ms: rho = -0.54, t(12) = -6.4, p<0.0001 for main dataset, rho = -0.38, t(14) = 1.2, p = 0.13). By contrast, the right occipitotemporal cluster was best explained by early layers in early time bins (80–110 ms: rho = -0.57, t(12) = -8.37, p<0.0001 for the main dataset and rho = -0.88, t(14) = 10.5, p<0.0001 for the replication dataset), then by mid-to-late-level layers between 120–200 ms (peak in layer 4 for main set, layer 5 for replication set; rho = 0.44, t(12) = 1.29, p = 0.11 for main; rho = -0.08, t(14)<1 for replication) and between 200–250 ms post stimulus onset (maximum in layer 6 for main set, maximum in layer 3 for replication set; rho = 0.17, t(12)<1). As evident in Fig 6, the later time bins did not have a significant rank-order correlation because of non-monotonic patterns between CNN layer and explained variability. Post-hoc t-tests (Benjamini-Hochberg corrected for multiple comparisons) revealed that for 120–200 ms, the numerical peak in layer 4 was statistically significant from both earlier layer 1 (t(12) = 4.78, p<0.001) and later layer 7 (t(12) = 3.66, p<0.001) and layer 8 (t(12) = 4.08, p<0.001). For 200–300 ms, the numerical peak in layer 6 was significantly different from layer 1 (t(12) = 5.17, p<0.0001) and layer 8 (t(12) = 3.3, p<0.005). Statistics for the replication set can be found in the Supporting Materials.

Similarly, while the frontal cluster best reflected lower layers of the CNN early 120–200 ms time bin (rho = -0.62, t(12) = -4.98, p<0.0005; see Supporting Materials for replication set), it best reflected information from the later layers, particularly the sixth CNN layer (FC6) in the 200–300 ms time bin (rho: 0.48, t(12)<1.) Post-hoc t-tests (corrected) revealed significantly more variance explained by layer 3 compared with layer 1 (t(12) = 2.29, p<0.05), and more in layer 6 compared with layer 7 (t(12) = 1.92, p<0.05) or layer 8 (t(12) = 1.87, p<0.05). Similarly, the replication dataset also had a local maximum of explained variance at the sixth CNN layer (see Supporting Materials for more information). Interestingly, the sixth layer had the maximum decodable category information (see Fig 2), suggesting that these signals reflect category-specific information. When comparing the latencies of maximum explained variability in these two clusters, we observed that the right occipitotemporal cluster peaked about 10 ms earlier than the frontal cluster (227 versus 236 ms). However, this difference was not statistically reliable (t(12)<1).

## Methods

### Ethics statement

Human subjects research was approved by the Colgate University Internal Review Board (IRB).

### Participants

Fourteen observers (6 female, 13 right-handed) participated in the study. One of the participant’s data contained fewer than half valid trials following artifact rejection and was removed from all analyses. An additional 15 participants (9 female, 12 right-handed) were recruited as part of an internal replication study (specific results from those participants are reported as Supporting Material). The age of all participants ranged from 18 to 22 (mean age = 19.1 for main experiment, and 19.4 for replication study). All participants had normal (or corrected to normal) vision as determined by standard ETCRS acuity charts. All participants gave Institutional Review Board-approved written informed consent before participating, and were compensated for their time.

### Stimuli

The stimuli consisted of 2250 color images of real-world photographs from 30 different scene categories (75 exemplars in each category) taken from the SUN database [24]. Categories were chosen to include 10 indoor categories, 10 urban categories, and 10 natural landscape categories, and were selected from the larger set of 908 categories from the SUN database on the basis of making maximally different RDMs when examining three different types of visual information (layer 7 features from a CNN, a bag-of-words object model, and a model of a scene’s functions, see [25] for more details on category selection). When possible, images were taken from the SUN database. In cases where this database did not have 75 images, we sampled from the internet (copyright-free images). Six of the 30 categories (bamboo forest, bar, butte, skyscraper, stadium, and volcano) were represented in the Places-205 database that comprised the training set for the CNN. Although the SUN and Places databases were designed to be complementary with few overlapping images [20], it is possible that some images in our set were included in the training set for the Places CNN. Care was taken to omit images with salient faces in them. All images had a resolution of 512 x 512 pixels (which subtended 20.8° of visual angle) and were processed to possess the same root-mean-square (RMS) contrast (luminance and color) as well as mean luminance. All images were fit with a circular linear edge-ramped window to obscure the square frame of the images, thereby uniformly distributing contrast changes around the circular edge of the stimulus [26,27].

### Apparatus

All stimuli were presented on a 23.6” VIEWPixx/EEG scanning LED-backlight LCD monitor with 1ms black-to-white pixel response time. Maximum luminance output of the display was 100 cd/m^2^, with a frame rate of 120 Hz and resolution of 1920 x 1080 pixels. Single pixels subtended 0.0406° of visual angle (i.e. 2.43 arc min.) as viewed from 32 cm. Head position was maintained with an Applied Science Laboratories (ASL) chin rest.

### Experimental procedure

Participants engaged in a 3 alternative forced-choice (3AFC) categorization task with each of the 2250 images. As it was not feasible for participants to view all images in one sitting, all images were randomly split into two sets, keeping roughly equal numbers of images within each category. Each image set was presented within a different ~50-minute recording session, run on separate days. The image set was counterbalanced across participants, and image order within each set was randomized. Participants viewed the windowed scenes against a mean luminance background under darkened conditions (i.e. the only light source in the testing chamber was the monitor). All trials began with a 500 ms fixation followed by a variable duration (500–750 ms) blank mean luminance screen to enable any fixation-driven activity to dissipate. Next, a scene image was presented for 750 ms followed by a variable 100–250 ms blank mean luminance screen, followed by a response screen consisting of the image’s category name and the names of two distractor categories presented laterally in random order (distractor category labels were randomly sampled from the set of 29 and therefore varied on a trial-by-trial basis). Observers selected their choice by using a mouse to click on the correct category name. Performance feedback was not given.

### EEG recording and processing

Continuous EEGs were recorded in a Faraday chamber using EGI’s Geodesic EEG acquisition system (GES 400) with Geodesic Hydrocel sensor nets consisting of a dense array of 256 channels (electrolytic sponges). The on-line reference was at the vertex (Cz), and the impedances were maintained below 50 kΩ (EGI amplifiers are high-impedance amplifiers–this value is optimized for this system). All EEG signals were amplified and sampled at 1000 Hz. The digitized EEG waveforms were band-pass filtered offline from 0.1 Hz to 45 Hz to remove the DC offset and eliminate 60 Hz line noise.

All continuous EEGs were divided into 850 ms epochs (100 ms before stimulus onset and the 750 ms of stimulus-driven data). Trials that contained eye movements or eye blinks during data epochs were excluded from analysis. Further, all epochs were subjected to algorithmic artifact rejection of voltage exceeding ± 100 μV. These trial rejection routines resulted in no more than 10% of the trials being rejected on a participant-by-participant basis. Each epoch was then re-referenced offline to the net average, and baseline corrected to the last 100 ms of the luminance blank interval that preceded the image. Grand average event-related potentials (ERPs) were assembled by averaging all re-referenced and baseline corrected epochs across participants. Topographic plots were generated for all experimental conditions using EEGLAB [28] version 13.4.4b in MATLAB (ver. R2016a, The MathWorks, MA).

For all analyses, we improved the signal-to-noise ratio of the single trial data by using a bootstrapping approach to build sub-averages across trials for each trial (e.g., [19]). Specifically, for each trial within a given scene category, we randomly selected 20% of the trials within that category and averaged those to yield a sub-averaged ERP for that trial. This was repeated until all valid trials within each category were built. This process was repeated separately for each participant. This approach is desirable as we are primarily interested in category-level neuroelectric signals that are time-locked to the stimulus.

### Deep convolutional neural network (CNN)

In order to assess the representations available in a deep convolutional neural network at each processing stage, we extracted the activations in each of eight layers in a pre-trained network. Specifically, we used a CNN based on the AlexNet architecture [29] that was pre-trained on the Places database [20] and implemented in Caffe [30]. The first five layers of this neural network are convolutional, and the last three are fully connected. The convolutional layers have three operations: convolution, pooling, and a rectified linear (ReLu) nonlinearity. For these layers, we extracted features after the convolution step. This CNN was chosen because it is optimized for 205-category scene classification, and because the eight-layer architecture, loosely inspired by biological principles, is most frequently used when comparing CNNs and brain activity [12,15,16]. For each layer, we averaged across images within a category, creating 30-category by N-feature matrices.

In order to assess the amount of category-related information available in each layer of the CNN, we performed a decoding procedure on the feature vectors from each CNN layer using a linear multi-class support vector machine (SVM) implemented as LIBSVM in Matlab [31]. Decoding accuracies for each layer were calculated using 5-fold cross validation.

For all analyses, statistical testing was done via permutation testing. Specifically, we fully exchanged row and column labels for the RDMs (1000 permutation samples per participant) to create an empirical chance distribution. To correct for multiple comparisons, we used cluster extent with a threshold of p<0.05, Bonferroni-corrected for multiple comparisons (similar to [12]).

### Time-resolved encoding analysis

A forward pass of activations through the CNN was collected for each image and each layer, and reshaped into a feature vector. Feature vectors were averaged across category to create eight 30-category by N-feature matrices. From these feature matrices, we created 30-category by 30-category correlation matrices. Representational dissimilarity matrices (RDMs) were created by transforming the correlation matrices into distance matrices using the metric of 1-Spearman rho [12,14]. Each RDM is a symmetrical matrix with an undefined diagonal. Therefore, in all subsequent analyses, we will use only the lower triangle of each RDM to represent patterns of category similarity.

To create neural RDMs, for each participant and for each electrode, we extracted ERP signals within a 40 ms sliding window beginning 100 ms before image presentation, and extending to the entire 750 ms image duration. For each 40 ms window, we created a 30 x 30 correlation matrix from the voltage values at that electrode, averaged over category. A 30 x 30 RDM using the same 1-minus-correlation distance metric described above. The window size was truncated at the end of each trial as to not extend beyond image presentation. Thus, RDMs were created from each of 256 electrodes for each of 850 ms time points. The upper and lower bounds of the noise ceiling for the data were computed as recommended in [21]. It is worth noting that while previous MEG RDM results have performed time-resolved analyses on a time point by time point manner taking the value at each sensor at each time point as a feature (e.g. [12]), our approach allows for the understanding of feature correspondence at each electrode, also enabling spatiotemporal analysis.

We computed a noise ceiling for the results following the method detailed in [14,21]. Briefly, the noise ceiling contains both lower- and upper-bound estimates of the group-average correlation with an RDM that would be predicted by an unknown true model. The upper bound consisted of the average correlation of each single participant to the group mean. As this includes all participants, it is overfit and therefore an overestimation of the true model’s fit. By contrast, the lower bound used a leave-one-participant out approach, computing each single-participant RDM’s correlation with the average RDM of the other participants. This avoids overfitting, but underestimates the true model’s average correlation due to the limited data.

### Electrode clustering

In order to examine spatial relations in encoding patterns across electrodes, we adopted a data-driven approach based on random field theory. We modified an algorithm initially created by Chauvin and colleagues used to assess statistical significance of pixel clusters in classification images [32]. This allowed us to identify spatially contiguous electrode clusters based on voltage differences, while remaining agnostic to any encoding differences with the CNN. Specifically, we submitted participant-averaged and z-transformed voltage difference topographic maps to the algorithm time point by time point. The spatial clustering algorithm then grouped electrodes that contained normalized voltage differences that significantly deviated from baseline noise (p < .001). As this spatial grouping procedure is based on assessing the statistically significant peaks and troughs of the voltage patterns at a given point of time, its advantage is that we did not need to set the number of clusters in advance. We selected clusters that persisted for more than 20 ms, resulting in five clusters: an early central occipital cluster (100–125 ms), an early central cluster (80–105 ms), two bilateral occipitotemporal clusters (70–500 ms), and a large frontal cluster (135–500 ms). We assessed the relative encoding strength of each of the eight CNN layers within each of these five clusters in all analyses.

## Discussion

In this work, we demonstrated that there is a substantial resemblance between the sequential scene category representations from each layer of a pre-trained deep convolutional neural network (CNN), and those in human ERP activity while observers are engaged in categorization. Early layers of the CNN best predicted early ERP activity, while later layers best predicted later activity. Furthermore, the total variability captured by the CNN was slightly less than half of the variability given by the noise ceiling of the data.

Furthermore, we observed a spatial correspondence between CNN layers and ERP variability. While electrodes over central- and left- occipitotemporal cortex were robustly predicted early by early CNN layers, electrodes over right occipitotemporal cortex had sequential representations that resembled the sequential representations of the CNN. Specifically, activity in these electrodes was best predicted by the second and third CNN layers before 100 ms post-stimulus, by the fourth layer between 120–200 ms, and by the sixth layer after 200 ms. A similar striking dissociation was observed over the frontal electrode cluster: early CNN layers best predicted early activity, but the more conceptual, fully-connected layers captured activity at the frontal electrodes about 100 ms later. Taken together, these results suggest a deeper homology between the human visual system and the deep neural network than the few biological principles that inspired the architecture [29]. This homology provides the unique intellectual opportunity to probe the mid- and late- stages of visual representation that have thus far been difficult to ascertain.

Because the CNN is an exclusively feedforward model, comparing the representations in the CNN and the human brain allows us to speculate on the extent to which human category representations are processed in a feedforward manner (such as [33]). We observed that earlier layers of the CNN explained more ERP variance compared with later layers of the CNN. This may be evidence that earlier visual processing is predominantly feedforward, while later visual processing requires feedback, consistent with other relatively early accounts of top-down feedback [22,34–36]. However, we also observed that the neural variability explained by the CNN only reached about half of the maximum possible explained variability given by the noise ceiling. This may suggest that feedback or recurrent processing plays a significant role in categorization [22,34,37]. Of course, it is possible that a different feedforward model, or a different weighting of features within this model may explain more variability in human ERP patterns. However, recent literature suggests that performance differences between different deep CNN architectures is smaller than the difference between CNNs and human observers, or between CNNs and previous models [14,15,25,38,39]. Future work will examine both recurrent and feedforward architectures to disentangle these possibilities.

In examining the residuals of the model fits, we found that the CNN over-estimated the similarity of natural landscape images while simultaneously under-estimating the similarity between different types of manufactured environments (indoor versus urban). This suggests a coarser-grained representation for natural landscape images in the CNN compared to human observers. This pattern may reflect the fact that only 29% of images and 28% of scene categories in Places-205 are natural landscape environments [20]. Having more training data for the CNN may have resulted in the ability to form finer-grained representations for manufactured environments.

Our results are largely in agreement with previous MEG studies that examined the correspondence between CNNs and time-resolved neural signals [12,18]. These studies examined whole-brain signals in a time point by time point manner, losing any spatial response pattern. By contrast, by using a sliding window on each electrode, we were able to retain the patterns across the scalp while still examining time-resolved data. Given the known representational differences between MEG and EEG [40], this work provides unique but complementary information about visual category representations. Specifically, [40] found that compared to EEG, MEG signals had decodable information earlier, and more driven by early visual cortex. This suggests that the time points reported here might constitute an upper bound on information availability.

A second difference between this work and previous is that we examined category-specific information instead of image-level information. As the act of recognition is generally an act of categorization [41], and because the CNN we used was pre-trained specifically to make category-level distinctions [42,43], we argue that this is the most natural comparison for human and artificial neural networks. Accordingly, we assessed the amount of category-level information available in each layer of the CNN. While it is unsurprising that significant category information exists in all eight layers, or that the amount of information increases across layers, we were surprised to observe that category information peaked in the sixth layer. Given that the utility of layer depth is still controversial within the computer vision community [44,45], this result may be of interest to this community as well. Furthermore, that both right occipitotemporal and frontal electrodes also had explained variability that peaked in the sixth CNN layer corroborates the view that a shallower artificial neural network might outperform deeper ones on scene classification tasks.

Although CNNs differ significantly from biological networks, they are of interest to neuroscientists because they allow us to see a solution to a difficult information-processing problem in a step-by-step manner. The extent to which hard problems such as visual recognition have unique solutions is an open question. Thus, the growing number of similarities between biological and neural networks may indicate that artificial neural networks have honed in on the same solution found by evolution and biology.

## Supporting information

S1 Fig Overall variance explained by all eight layers of the CNN for the supplementary dataset. (EPS)

S2 Fig Overall variance explained by all eight layers of the CNN as a function of electrode group. (EPS)

S3 Fig Variance explained by each layer of the CNN alone. As in Fig 5 in the main text, CNN layers are ordered darkest (Conv 1) to lightest (FC8).
(EPS)

S4 Fig Onset of significant explained variance as a function of CNN layer. (EPS)

S5 Fig Latency of maximum explained variability as a function of CNN layer for replication dataset. (EPS)

S6 Fig Residuals across superordinate category for replication set. (EPS)

S7 Fig Explained variability in frontal cluster between 200–300 ms after stimulus onset. Each trace reflects an individual participant.
(EPS)

S1 Text Text of supporting information, part 1. (DOCX)

S2 Text Text of supporting information, part 2. (DOCX)
