# Supplementary material for: Shared spatiotemporal category representations in biological and artificial deep neural networks
Source: PLoS Comput Biol. 2018 Jul 24;14(7):e1006327. doi: 10.1371/journal.pcbi.1006327 (PMC6075788; doi:10.1371/journal.pcbi.1006327)
Supplement: S1 Text — (DOCX) [file pcbi.1006327.s008.docx]

**Supporting Information Text, part 1**

Here, we report the results of an internal replication experiment based on the experimental procedure reported in the main article. The experimental procedure was identical to the primary experiment, with the only exception being that the scene stimuli were presented to 500 msec. Likewise, all recording parameters and post-processing routines were identical to those reported in the primary experiment excepting that the EEG waveforms were segmented 100 ms before stimulus onset and 500 ms following stimulus onset (i.e., 600 ms epochs). Figure S1 shows the variability explained over time by all of the eight CNN layers over time.
