# Supplementary material for: Shared spatiotemporal category representations in biological and artificial deep neural networks
Source: PLoS Comput Biol. 2018 Jul 24;14(7):e1006327. doi: 10.1371/journal.pcbi.1006327 (PMC6075788; doi:10.1371/journal.pcbi.1006327)
Supplement: S2 Text — (DOCX) [file pcbi.1006327.s009.docx]

**Supporting text, part 7**

**Five cluster analysis: frontal cluster**

Between 120-200 ms, we observed that the third CNN layer had the maximum explained variance in the frontal cluster. Post-hoc t-tests, corrected for multiple comparisons with the Benjamini-Hochberg procedure, revealed that the explained variability was greater in Layer 3 than in Layer 1 (t(14)=2.77, p<0.008), and also more than Layer 8 (t(14)=2.89, p<0.006), or Layer 7 (t(14)=2.75, p<0.008). For 200-300 ms, we observed a near bimodal distribution of explained variability that was largest for Layer 1, but also visible in all participants in Layer 6 (see Figure S7). A post-hoc paired t-test revealed that there was significantly greater explained variability in Layer 6 compared with Layer 5 (t(14)=-9.2, p<0.0001) even through the overall rank-order correlation between layer and explained variability was significantly negative, unlike what was observed in the main dataset (rho=-0.81, t(14)=3.92, p<0.001).
